# Guideline on anterior cruciate ligament injury: A multidisciplinary review by the Dutch Orthopaedic Association

**DOI:** 10.3109/17453674.2012.704563

**Published:** 2012-08-25

**Authors:** Duncan E Meuffels, Michelle T Poldervaart, Ron L Diercks, Alex WFM Fievez, Thomas W Patt, Cor P van Hart, Eric R Hammacher, Fred van Meer, Edwin A Goedhart, Anton F Lenssen, Sabrina B Muller-Ploeger, Margreet A Pols, Daniel B F Saris

**Affiliations:** ^1^The Dutch Orthopaedic Association (Nederlandse Orthopaedische Vereniging (NOV)); ^2^The Dutch Society for Arthroscopy (Nederlandse Vereniging voor Arthroscopie (NVA)); ^3^The Association of Surgeons of the Netherlands (Nederlandse Vereniging voor Heelkunde (NVvH)); ^4^The Dutch Society of Rehabilitation (Vereniging van Revalidatieartsen (VRA)); ^5^The Society for Sports Medicine (Vereniging voor Sportgeneeskunde (VSG)); ^6^The Royal Dutch Society for Physiotherapy (Koninklijke Genootschap voor Fysiotherapie (KNGF)); ^7^The Department of Professional Quality, the Dutch Association of Medical Specialists (Orde van Medisch Specialisten), the Netherlands

## Abstract

The Dutch Orthopaedic Association has a long tradition of development of practical clinical guidelines. Here we present the recommendations from the multidisciplinary clinical guideline working group for anterior cruciate ligament injury.

The following 8 clinical questions were formulated by a steering group of the Dutch Orthopaedic Association.

- What is the role of physical examination and additional diagnostic tools?
- Which patient-related outcome measures should be used?
- What are the relevant parameters that influence the indication for an ACL reconstruction?
- Which findings or complaints are predictive of a bad result of an ACL injury treatment?
- What is the optimal timing for surgery for an ACL injury?
- What is the outcome of different conservative treatment modalities?
- Which kind of graft gives the best result in an ACL reconstruction?
- What is the optimal postoperative treatment concerning rehabilitation, resumption of sports, and physiotherapy?

These 8 questions were answered and recommendations were made, using the “Appraisal of Guidelines for Research and Evaluation” instrument. This instrument seeks to improve the quality and effectiveness of clinical practical guidelines by establishing a shared framework to develop, report, and assess. The steering group has also developed 7 internal indicators to aid in measuring and enhancing the quality of the treatment of patients with an ACL injury, for use in a hospital or practice.

Anterior cruciate ligament injury is a common sports injury with a worldwide reconstruction rate of more than 200,000 per year (Meuffels et al. 2011). Clinically practical guidelines have been used for a long time; the Dutch Orthopaedic Association has a long experience of guideline development, since the 1980s. This is the recommendation from the multidisciplinary clinical guideline “anterior cruciate ligament injury”, set up and aimed at all the members of the medical disciplines concerned with diagnosis and treatment of anterior cruciate ligament injury. This injury is seen by a large number of diverse medical caregivers, and the importance of a team approach to injury treatment with a view to reintegration in sport has been established. This guideline was set up using the “Appraisal of Guidelines for Research and Evaluation (AGREE)” instrument (www.agreecollaboration.org).

## Methods

The process started with the formulation of 8 clinical questions by a steering group of the Dutch Orthopaedic Association.

### Literature search

The guideline was meant to cover fully-grown adolescents up to active patients of middle-age. A general search was performed for existing guidelines in the databases of National Guideline Clearinghouse (http://www.guideline.gov/), NICE (http://www.nice.org.uk/), SIGN (http://www.sign.ac.uk/), CBO (http://www.cbo.nl/thema/Richtlijnen/), and using the search machine SUMSearch (http://sumsearch.uthscsa.edu/). We also searched for systematic reviews in the Cochrane Library (http://www.cochrane.org). For each question, the bibliographic database Medline (OVID) (1950-2010) was searched for specific terms (Table 1, see supplementary data). We searched for randomized trials and systematic reviews or meta-analyses. If none were found, a broader search of studies of a lower level of evidence was performed, including case-control studies and cohort studies (both prospective and retrospective). Afterwards, a hand search was performed using the reference lists of the previously found articles.

### Grading of study quality

After selection of the relevant literature, the members of the steering group and a methodologist graded the studies for levels of evidence and quality (Table 2). For each query, the scientific level of evidence was graded and the conclusion was summarized (Table 3).

**Table 2.** Level of evidence of the conclusion

Level of evidence	Interventional studies	Diagnostic accuracy studies	Harm, side effects, etiology, prognosis
A1	Systematic review/meta-analysis of at least 2 independently conducted studies of A2 level.		
A2	Randomized, double blind trial with good study quality and an adequate number of study participants.	Index test compared to reference test (reference standard); cut-offs were defined a-priori; independent interpretation of test results; an adequate number of consecutive patients were enrolled; all patients received both tests.	Prospective cohort study of sufficient magnitude and follow-up, adequately controlled for “confounding” and no selective follow-up.
B	Clinical trial, but without all the features mentioned for level A2 (including case-control study, cohort study).	Index test compared to reference test, but without all the features mentioned for level A2.	Prospective cohort study, but without all the features mentioned for level A2 or retrospective cohort study or case-control study.
C	Non-comparative studies.		
D	Expert opinion.		

**Table 3.** Level of evidence of the conclusion

Level	Conclusion based on:
1	A1 study or at least 2 independent studies of level A2.
2	1 study of level A2 or at least 2 independent studies of level B.
3	1 study of level B or C.
4	Expert opinion.

**Table 4.** Internal indicators for ACL injury

Type of indicator	Operationalization
Structural	Is the KOOS and/or IKDC subjective used in the treatment process?
Process	What is the percentage of patients registered with an outcome scored with KOOS and/or IKDC?
Structural	Is there a protocol for MRI, based on the ACL guideline, that indicates when imaging is necessary?
Structural	Is there a rehabilitation protocol encompassing sensomotoric training, open- and closed-chain strength training, and optimization of range of motion?
Structural	Has the rehabilitation protocol been updated at least every 3 years?
Outcome	What is the median time between the occurrence of the ACL insufficiency and the operation?
Process	What is the percentage of patients who have been operated on within a year of the ACL insufficiency occurring?

### Recommendations

The recommendations given are influenced by many considerations apart from the scientific evidence—such as patient preferences, availability of facilities, or organizational aspects. The recommendations for each question have been based on the scientific evidence, combined with the most important considerations, such as input from the patient focus group and feedback from the participating medical societies.

## Questions addressed in the guideline

### What is the role of physical examination and additional diagnostic tools?

***Scientific evidence***

***Level 1:***

The Lachman test is the most valid stability test at the physical examination of the knee, with a sensitivity of 85% and a specificity of 95% (Solomon et al. 2001, Scholten et al. 2003, Benjaminse et al. 2006).

Performance of a complete physical examination of the knee (Lachman test, pivot shift, anterior drawer test) has a higher sensitivity and specificity than a partial investigation (Solomon et al. 2001).

MRI is a valid and safe non-invasive diagnostic tool for diagnosing anterior cruciate ligament injury, with a high sensitivity and specificity (both 94%) (Oei et al. 2003, Crawford et al. 2007).

***Level 2:***

It is likely that, when physical examination is conducted well, an MRI has no added value, since it will seldom change the diagnosis or the treatment strategy (Liu et al. 1995, Gelb et al. 1996, Kocabey et al. 2004).

*Recommendation.* In order to maximise the diagnostic accuracy for an anterior cruciate ligament injury, it is recommended that the Lachman test, pivot shift test, and anterior drawer test of the knee be performed. Having an experienced investigator enhances the reliability of this physical examination.

MRI has no additional value when physical examination has shown anterior-posterior or rotational instability of the knee, suggesting an anterior cruciate ligament injury. However, MRI is a reliable additional investigation to establish other intraarticular lesions.

### Which patient-related outcome measures should be used for the evaluation and follow-up of patients with anterior cruciate ligament injury?

***Scientific evidence***

***Level 1:***

Performance of a complete physical examination of the knee (Lachman, pivot shift, and anterior drawer test) has a higher sensitivity and specificity than performing a partial examination (Solomon et al. 2001, Scholten et al. 2003, Benjaminse et al. 2006).

***Level 2:***

The IKDC and KOOS are validated (in Dutch) (Haverkamp et al. 2006, de Groot et al. 2008) as patient-related outcome scores. These knee-related scores are probably well-suited for patients with an ACL rupture (Roos et al. 1998, Irrgang et al. 2001).

The Tegner score is an accepted activity score (Briggs et al. 2009); it is has not, however, been validated in Dutch.

*Recommendation. *We recommend the combination of the Lachman test, pivot shift test, and anterior drawer test as a clinical outcome measurement. We recommend use of the IKDC subjective and the KOOS as patient-related outcome measures. It can be useful to adopt the Tegner score as an outcome measurement for activity.

### What are the relevant parameters that influence the indication for an anterior cruciate ligament reconstruction?

***Scientific evidence***

***Level 1:***

Actual age is not a factor of importance for the decision to perform an ACL reconstruction (Barber et al. 1996, Sloane et al. 2002).

Younger patients are entitled to an ACL reconstruction earlier because of their higher activity level (Barber et al. 1996, Ferrari and Bach 2001, Sloane et al. 2002, Dunn et al. 2004).

***Level 3:***

The activity level of the patient is probably the most important predictor for the necessity to perform an ACL reconstruction. The more the patient is active in pivoting sports, the greater the chance that an operation is necessary to reach an acceptable activity level (Daniel and Fithian 1994).

Reconstruction of the ruptured ACL might reduce the chance of further meniscal and/or cartilage damage (Dunn et al. 2004).

*Consideration.* Timing of the operative procedure is an important issue. The reconstruction should be performed at the time that the knee function has been optimized, and the synovial reaction has quietened down. Other considerations such as cartilage damage or degeneration can influence the choice of an operative procedure. From a patient’s point of view, other non-medical motives can play an important role. Professional or upcoming talented sports people may have different expectations and wishes considering operative or conservative treatment of an ACL rupture.

*Recommendation.* If symptomatic instability of the knee, as a result of an anterior cruciate ligament injury, is not reduced after physiotherapy nor after adjustment of activity, anterior cruciate ligament reconstruction is recommended. This might prevent multiple interventions because of further meniscal and cartilage damage.

In adults, when deciding between nonoperative or operative treatment, age should not be weighed as an important factor.

In children, it may be preferable to await surgery until the growth plates are (almost) closed.

An anterior cruciate ligament reconstruction should only be performed in a “quiet” knee with a normal range of motion.

### Which findings or complaints are predictive of a bad result of an anterior cruciate ligament injury treatment?

***Scientific evidence***

***Level 2:***

A longer period between the occurrence of the ACL rupture and the reconstruction could increase the risk of meniscal and/or cartilage damage (Fithian et al. 2005, Gregory and Landreau 2008, Joseph et al. 2008, Slauterbeck et al. 2009).

***Level 3:***

Persistent subjective knee instability has a negative influence on the outcome of both nonoperative and operative treatment. Treatment outcome is negatively influenced by undergoing multiple knee interventions of any kind (Meunier et al. 2007).

An extension deficit before the operation can have a negative effect on the outcome of an ACL reconstruction (Mauro et al. 2008).

A strength deficit of more than 20% of the hamstring and quadriceps muscles compared to the uninjured side can have a negative effect on the outcome of an ACL reconstruction (de Jong et al. 2007, Eitzen et al. 2009).

Cartilage and/or meniscal damage can have a negative effect on the functional result of the treatment of an ACL injury (Williams et al. 2000, Meunier et al. 2007, Kowalchuck et al. 2009).

Continued participation in “high-risk sports” predisposes the knee for injury of the cartilage, the meniscus, and the possibly reconstructed ACL—increasing the risk of re-rupture, secondary surgery, and knee osteoarthritis (Fink et al. 2001, Salmon et al. 2005, Meuffels et al. 2009). There is insufficient evidence to prove the protective effect of an ACL reconstruction against knee osteoarthritis (Fithian et al. 2005, Gregory et al. 2008, Joseph et al. 2008, Meuffels et al 2009, Slauterbeck et al. 2009).

Leg malalignment could have a negative influence on the outcome of an ACL reconstruction. Combining an ACL reconstruction and a correcting osteotomy could make the outcome of the ACL reconstruction more predictable (Williams et al. 2000).

There is no clear evidence to show that the patient’s gender influences the outcome of an ACL reconstruction (Salmon et al. 2005, Heijne et al. 2008, Slauterbeck et al 2009).

*Consideration.* From a patient’s point of view, the definition of a “bad result” may differ from the specific medical-technical definition. It is important to give clear counseling about the expected activity level in both the short and long term. The uncertainty of a nonoperative treatment can be more difficult to accept for a sports person at a high level than for a person who is more interested in sport for recreation, or an elderly patient. One should also take the working circumstances of the person involved into consideration.

*Recommendation.* An anterior cruciate ligament reconstruction should be performed only when a full extension of the knee is possible and the synovial reaction is minimal.

During the preoperative preparations, a possible muscle strength deficit of the injured leg should be treated.

In the presence of knee malalignment and anterior cruciate ligament insufficiency, correction of the leg alignment should be considered, possibly in combination with an anterior cruciate ligament reconstruction.

It is recommended that the patient be informed that participation in high-risk sports or heavy knee labor increases the risk of cartilage damage, meniscal damage, and damage to the reconstructed anterior cruciate ligament, which could result in a re-rupture, secondary surgery, or knee osteoarthritis.

### What is the optimal timing for surgery for an anterior cruciate ligament injury?

***Scientific evidence***

***Level 2:***

The increase in time between the injury and reconstruction of the ACL is a risk factor for meniscal and cartilage damage (Church and Keating 2005, Foster et al. 2005, Kim et al. 2005, Vasara et al. 2005. Seon et al. 2006, Ohly et al. 2007, Papagasteriou et al. 2007, Granan et al. 2009, Tayton et al. 2009 Vasara et al. 2005).

***Level 3:***

At long-term follow-up (7 years) of a subacute reconstruction (within 6 weeks) gave better outcome for range of motion, work participation, and degenerative change than late reconstruction (Järvelä et al. 1999).

*Recommendation.* The indication for a reconstruction is persistent instability of the knee with complaints of giving way. This diagnosis is difficult to make in an acute situation. We therefore recommend that anterior cruciate ligament reconstruction should not be performed in the first weeks after trauma, in order to minimize the risk of operating on an asymptomatic patient.

If the indication for anterior cruciate ligament reconstruction has been defined, we recommend performing the reconstruction in a timely manner in order to minimize the risk of additional damage to the cartilage and/or meniscus.

The patient with a delayed reconstruction (6 weeks to 3 months post-trauma) can resume his or her physical activity sooner—with a greater chance of obtaining higher activity scores—than a patient with a late reconstruction (more than 3 months after trauma).

In the long term, delayed reconstruction gives a better range of motion and less degenerative changes than a late reconstruction.

### What is the outcome of different non-operative treatment modalities?

***Scientific evidence***

***Level 1:***

Balance and proprioception training has a positive effect on joint position sense, muscle strength, experienced knee function, outcome of functional capacity, and return to full activity (Fitzgerald et al. 2000, Cooper et al. 2005, Trees et al. 2005, 2007).

***Level 2:***

Addition of open-chain strength training to an ACL rehabilitation program has a positive effect on muscle strength of quadriceps and hamstring muscles and on functional recovery (Zatterstrom et al. 2000, Perry et al. 2005, Tagesson et al. 2008).

Supervised training has more value than non-supervised training concerning muscle strength of the quadriceps and hamstring muscles, and on functional recovery (Zatterstrom et al. 1998, 2000).

***Level 3:***

The sensation of instability is reduced for ACL-injured individuals by wearing a knee brace, but initially, the use of a brace can also lead to more complaints in activities of daily living (Swirtun et al. 2005).

*Recommendation.* It is advisable to rehabilitate patients with an anterior cruciate ligament injury using a physiotherapy exercise program that trains multiple ground-motoric abilities.

We strongly recommend incorporating senso-motoric training (balance and proprioception) into the rehabilitation program.

It is preferable to incorporate both open- and closed-chain strength training into the rehabilitation program after an anterior cruciate ligament injury.

There are no indications for use of a brace in the standard treatment of an ACL injury.

A brace could be considered for patients with instability, who do not qualify or who do not want to qualify for operative treatment.

### Surgical treatment— which kind of graft gives the best result in an anterior cruciate ligament reconstruction?

***Scientific evidence***

***Level 1:***

Bone-patella-tendon-bone and hamstring grafts both give similar degrees of stability when used in conjunction with modern (extra-cortical) fixation techniques (Schultz and Carr 2002, Goldblatt et al. 2005, Prodromos et al. 2005, Thompson et al. 2005).

The use of a bone-patella-tendon-bone autograft has a greater chance of giving anterior knee pain than the use of a hamstring autograft. There is no substantial difference between hamstring or bone-patella-tendon-bone reconstruction, in muscle strength of the flexors and extensors of the knee 2 years after surgery (Freedman et al. 2003, Dauty et al. 2005, Forster and Forster 2005, Goldblatt et al. 2005).

***Level 2:***

There is no significant clinical difference between allograft and autograft ACL reconstruction in IKDC, activity scores, and stability (Carey et al. 2009, Krych et al. 2008, Sun et al. 2009).

Radiating the allograft can give higher failure rates. Pre-tensioning of the allograft before the reconstruction has no additional value (Ejerhed et al. 2001, Gorschewski et al. 2005, Rappe et al. 2007, Sun et al. 2009).

At short-term follow-up (2 years), there is no difference in patient-related outcome between single- and double-bundle ACL reconstruction. At short-term follow-up, there is a better recovery of the rotational stability when performing a double-bundle reconstruction (Kondo et al. 2008, Meredick et al. 2008, Seon et al. 2008, Siebold and Zantop 2008, Streich et al. 2008, Tsuda et al. 2009, Wang et al. 2009).

Suturing of ACL ruptures does not lead to good results; there is an increased chance of knee osteoarthritis and many patients report knee instability and ruptures (25–30% after 5 years) (Engebretsen et al. 1989).

Enhancement of the graft with, for example, a Kennedy LAD does not increase stability, diminish ruptures, or improve function, but it does lead to more side effects (swelling, infection, and need for revision) (Grontvedt et al. 1995, Drogset and Grontvedt 2002, Muren et al. 2003).

Use of a synthetic graft (Leeds-Keio, Gore-Tex) leads to more instability, more ruptures, more pain, and lower activity scores (Engebretsen et al. 1989, Engstrom et al. 1993, Grontvedt et al. 1995, 1996, Drogset and Grontvedt 2002, Muren et al. 2003).

***Level 3:***

In different modern methods using metal or resorbable screws, graft fixation strength is similar (Brand et al. 2000, Harvey et al. 2005).

*Recommendation.* Considering clinical outcome measurement, there is no direct preference for the use of either autograft or allograft for anterior cruciate ligament reconstruction. Both graft types lead to good clinical results.

Radiated allografts fail more often than non-radiated allografts.

Stretching of allografts before the reconstruction is unnecessary.

Bone-patellar-tendon-bone and hamstring reconstructions give good results, stability, and low complication rates. Hamstring reconstruction results in significantly less anterior knee pain. Both single- and double-bundle hamstring reconstruction give good functional results. With our current scientific knowledge, there is no preference for either technique. Double-bundle reconstruction is a more time consuming and technically more demanding procedure than single-bundle reconstruction.

Use of synthetic graft or ligament augmentation is not recommended because of inferior results and increased complications in long-term follow-up.

There is no scientific basis for making recommendations as to the choice of type of fixation device for the different grafts.

### What is the optimal postoperative treatment (after the first postoperative check-up, concerning rehabilitation, resumption of sports, and physiotherapy)?

***Scientific evidence***

***Level 1:***

Wearing of a knee brace has no additional treatment value after an ACL reconstruction (Wright and Fetzer 2007, Anderson et al. 2009).

In the early phase of rehabilitation, closed-chain exercise therapy is likely to give fewer patello-femoral complaints and less laxity than open-chain exercises (Trees et al. 2005, Wright et al. 2008, Anderson et al. 2009).

***Level 2:***

Addition of neuromuscular training to the rehabilitation program will have a better outcome than strength training alone (Risberg et al. 2007).

An exercise program with early open-chain exercises (4 weeks postoperatively) will lead to more laxity with hamstring grafts than late open-chain exercises (12 weeks postoperatively) (Heijne and Werner 2007).

*Consideration.* The literature retrieved gives insufficient scientific information for us to be able to give advice concerning work, daily living, and resumption of sports that can be applied to every patient. On every occasion of the rehabilitation program, the treatment team should be aware of signals such as knee pain, swelling, feeling of warmth, and range of motion. With this information, an individual schedule can be implemented concerning daily living, work, and sports to ensure a swift and safe rehabilitation.

*Recommendation.* We recommend combining strength with neuromuscular training in the postoperative treatment.

It is recommended that only closed-chain exercises be used in the early rehabilitation phase.

There is no reason for the use of a brace in the postoperative treatment period after an anterior cruciate ligament reconstruction.

Heavy physical activity in labor or sports should not be resumed within 3 months of surgery.

### Indicators

The steering group has developed 7 internal indicators to aid in measuring and enhancing the quality of the treatment of patients with an ACL injury, for use in a hospital or medical practice. They are summarized in Table 4. These indicators were not developed for use as an external quality control (external indicators).

## Supplementary data

Table 1 is available on the website (www.actaorthop.org), identification number 5465.
