# Supplementary material for: Guideline on anterior cruciate ligament injury: A multidisciplinary review by the Dutch Orthopaedic Association
Source: Acta Orthop. 2012 Aug 25;83(4):379–86. doi: 10.3109/17453674.2012.704563 (PMC3427629; doi:10.3109/17453674.2012.704563)
Supplement: Supplementary file 1 [file ORT-1745-3674-83-379-s5465.pdf]

Supplementary article data

Guideline on anterior cruciate ligament injury

A multidisciplinary review by the Dutch Orthopaedic Association

Duncan E Meuffels[1], Michelle T Poldervaart[1], Ron L Diercks[1], Alex WFM Fievez[1], Thomas W Patt[1], Cor P van der Hart[2], Eric R Hammacher[3], Fred van der Meer[4], Edwin A Goedhart[5], Anton F Lenssen[6], Sabrina B Muller-Ploeger[7], Margreet A Pols[7], and Daniel B F Saris[1]

[1]The Dutch Orthopaedic Association (Nederlandse Orthopaedische Vereniging (NOV)); [2]the Dutch Society for Arthroscopy (Nederlandse Vereniging voor Arthroscopie (NVA)); [3]the Association of Surgeons of the Netherlands (Nederlandse Vereniging voor Heelkunde (NVvH)); [4]the Dutch Society of Rehabilitation (Vereniging van Revalidatieartsen (VRA)); [5]the Society for Sports Medicine (Vereniging voor Sportgeneeskunde (VSG)); [6]the Royal Dutch Society for Physiotherapy (Koninklijke Genootschap voor Fysiotherapie (KNGF)); [7]the Department of Professional Quality, the Dutch Association of Medical Specialists (Orde van Medisch Specialisten), the Netherlands
Correspondence: d.meuffels@erasmusmc.nl
Submitted 12-02-27. Accepted 12-04-15

Tabel 1. Search strategy

Question	Database	Search strategy
1. Patient related outcome measures for the evaluation and follow-up of patients with ACL injury.	Medline (OVID) 1950-okt.2009	1. ("patient outcome" or pain or VAS or "muscle strength" or "functional outcome" or "Range of Motion" or " Sport*" or Participation or ICF or Tegner or "hop test" or "gait analysis" or "Laxity" or "KT 1000" or Kneelax or laxity or "tunnel placement" or "International knee documentation committee" or KOOS or Lysholm or "knee rating score" or "Marshall HSS score" or "SF 36"). ab,ti.<br>2. exp *"Outcome Assessment (Health Care)"/ or outcome measurement.mp.<br>3. (outcomes or outcome measure*).ab,ti.<br>4. "Quality of Life"/<br>5. Rehabilitation, Vocational/<br>6. Questionnaires/<br>7. 11 or 13 or 10 or 9 or 12 or 14<br>8. "Reproducibility of Results"/<br>9. 15 and 16
2. Which findings or complaints are predictive of a bad result of an ACL injury treatment?	Medline (OVID) 1950-dec. 2009	1. exp *Prognosis/<br>2. (prognostis or prognostic or predicti*).ab,ti.<br>3. Patient Selection/<br>4. patient selection.m_titl.<br>5. or/13-16<br>6.. (retrospective* or prospective*).ab,ti.<br>7. (predicti* or prognos*).ti.<br>8 6 or 7<br>9. 8 and 5<br>10. limit 9 to (animals and yr="1999 -Current")<br>11. 9 not 10<br>12. exp Epidemiologic Studies/ (zie fig.1)<br>13. 11 and 12<br>14. limit 13 to (clinical trial, all or clinical trial, phase iii or clinical trial, phase iv or clinical trial or comparative study or consensus development conference or consensus development conference, nih or controlled clinical trial or evaluation studies or government publications or guideline or meta analysis or multicenter study or practice guideline or randomized controlled trial or research support, nih, extramural or research support, nih, intramural or research support, non us gov't or research support, us gov't, non phs or research support, us gov't, phs or review or technical report or validation studies)<br>32. 13 or 14<br>Also limited for systematic review and RCT.

Open Access - This article is distributed under the terms of the Creative Commons Attribution Noncommercial License which permits any noncommercial use, distribution, and reproduction in any medium, provided the source is credited.
DOI 10.3109/17453674.2012.704563

3. What is the role of physical examination and additional diagnostic tools?	PubMed<br>1950-okt. 2009<br><br>Dutch, English, German.	1. exp Anterior Cruciate Ligament/in [Injuries]<br>2. (rupture* or injur*).ab,ti.<br>3. ACL.m_titl.<br>4. anterior cruciate ligament.ab,ti.<br>5. 1 or 3 or 4<br>6. 2 and 5<br>7. 5 or 6<br>8. *Knee Injuries/di, cl [Diagnosis, Classification]<br>9. 8 or 7<br>10. exp Magnetic Resonance Imaging/<br>11. 8 or 9<br>12. 9 and 11<br>13 ”Sensitivity and Specificity”/<br>14. ”Predictive Value of Tests”/<br>15. (sensitivity or specificity or agreement or kappa or predict*).ab,ti.<br>18. or/13-15<br>19. 18 and 12<br>20. limit 19 to animals<br>21. 19 not 20<br>22. limit 31 to (dutch or english or german)<br>23. limit 22 to (clinical conference or clinical trial or comparative study or consensus development conference or consensus development conference, nih or controlled clinical trial or evaluation studies or government publications or guideline or meta analysis or multicenter study or practice guideline or randomized controlled trial or research support, nih, extramural or research support, nih, intramural or research support, non us gov’t or research support, us gov’t, non phs or research support, us gov’t, phs or ”review” or technical report or validation studies)
4. What are the relevant parameters that influence the indication for an ACL reconstruction?	PubMed<br>2000 – okt. 2009<br>English, Dutch, German, French.	1. Age Factors/<br>2. Sex Factors/<br>3. Body Mass Index/<br>4. “Severity of Illness Index”/<br>5. Comorbidity/<br>6. Pregnancy/<br>7. Risk Factors/ or (gender or age or trauma or pregnancy).ab,ti.<br>8. exp Epidemiologic Factors/<br>9 or/ 1-8<br>10. exp Treatment Outcome/<br>11. “effect*”.af.<br>12. 10 or 11<br>13. 9 and 12<br>15. (indication* or contra-indication* or contraindication* or management).ti.<br>16 “guideline*”.af.<br>17. “algorithm*”.ti.<br>18. or /15-16<br>19. 13 and 18<br>20. Limit 19 clinical trial, all or clinical trial or comparative study or consensus development conference or consensus development conference, nih or controlled clinical trial or government publications or guideline or meta analysis or multicenter study or practice guideline or randomized controlled trial or research support, nih, extramural or research support, nih, intramural or research support, non us gov’t or research support, us gov’t, non phs or research support, us gov’t, phs or “review” or validation studies<br>21. exp epidemiologic studies/ or exp clinical trials as topic/ or intervention studies/<br>22. 20 and 19<br>21. 20 or 22

5. What is the outcome of different conservative treatment modalities?	Medline (Ovid)<br>1995-okt. 2009<br>English, Dutch, German.	1.. "physical therap* ".m_titl.<br>2. Physical Therapy Modalities/<br>3. Gait/<br>4. training.ab,ti.<br>5. exercise.ab,ti.<br>6. rehabilitation.ab,ti.<br>7. exp Rehabilitation/<br>8. Braces/<br>9. (bracing or brace*).m_titl.<br>10. "physical therap* ".m_titl.<br>11. Physical Therapy Modalities/<br>12. Gait/<br>13. training.ab,ti.<br>14. exercise.ab,ti.<br>15 conservative.ab.ti.
6. What is the optimal timing for surgery for an ACL injury?	Medline (OVID)<br>Embase<br>1950-okt. 2009<br>English, Dutch, German.	1. (early phase or late phase).ab,ti.<br>2. early.ab,ti.<br>3. timing.ab,ti.<br>4. epidemiologic studies/ or prospective studies/ or intervention studies/<br><br>MESH-term time factors did not provide adequate titels.
7. Which kind of graft gives the best result in an ACL reconstruction?	Medline (OVID)<br>1950-dec. 2009<br>English, Dutch, German.	**ACL Patella vs Hamstring**<br>1. Tendons/tr [Transplantation]<br>2. Transplantation, Autologous/<br>3. Tendon Transfer/<br>4. 1 or 3<br>5. (patellar tendon or hamstring tendon).ab,ti.<br>6 4 and 5<br>7. (patellar adj2 tendon).ab,ti.<br>8. BPTB.ab,ti.<br>9. (bone adj2 tendon).ab,ti.<br>10. (hamstring adj2 tendon).ab,ti.<br>11. 4SHS.ab,ti.<br>12. hamstring.ab,ti.<br>13 or/ 7-13<br>31. 29 and 30<br><br>**ACL Autograft_allograft**<br>1. (allograft* or autograft*).ab,ti.<br><br>**Synthetic**<br>1. synthetic.m_titl.<br>2.. "Polyethylene Terephthalates"/<br>3.. "Biocompatible Materials"/<br>4. "Prostheses and Implants"/<br>5. Implants, Experimental/<br>6. "Carbon"/<br>7. polyester.ti.<br>8. polyethylene.ti.<br>9. plastic.ti.<br>10. (polyester adj3 graft$).ab,ti.<br>11. (plastic adj3 graft$).ab,ti.<br>12. (synthetic adj3 graft$).ab,ti.<br>13. (polyethylene adj3 graft$).ab,ti.<br>14. (artificial adj3 graft).ab,ti.<br>15. (Gore-tex or Leeds-Keio or Kennedy LAD or Ligament augmentation device or PDS).ab,ti.

8. What is the optimal post-operative treatment?	Medline (OVID) 1995-dec. 2009 English, Dutch, German.	1. “physical therap* “.m_titl.<br>2. exp Physical Therapy Modalities/<br>3. Gait/<br>4. gait training.ab,ti.<br>5. (exercise* or brace* or bracing).ti.<br>6. or/1-5<br>8. rehabilitation.ti.<br>9. exp Rehabilitation/<br>10. postoperative.ti.<br>11. postoperative care/ or postoperative period/<br>12. after.m_titl.<br>13. (“return to sport” or “return to work”).af.<br>14. or/8-13<br>15. 14 and 6
